# Approaches to the Prevention and Treatment of Postpartum Hemorrhage: A Systematic Review of Past Advances, Recent Developments, and Best Practices

**DOI:** 10.7759/cureus.65096

**Published:** 2024-07-22

**Authors:** Oluwatosin D Sadiku, Susan A Aina, Chinonso C Odoemene, Temiloluwa E Ogunmoyin, Victor O Adedara, Omolara Olasimbo, Faridah A Ashir, Stephennie C Adili, Azeez O Kuteyi, Opeyemi O Fakayode, Donald O Faletti, María Belén Nogales Bernal

**Affiliations:** 1 Obstetrics and Gynecology, St. George's University School of Medicine, St. George's, GRD; 2 Medicine, St. George's University School of Medicine, St. George's, GRD; 3 Medicine, Family Medicine, and Obstetrics, St. George's University School of Medicine, St. George's, GRD; 4 Internal Medicine and Neurology, St. George's University School of Medicine, St. George's, GRD; 5 Internal Medicine, Temple University Hospital, Philadelphia, USA; 6 Internal Medicine, St. George's University School of Medicine, St. George's, GRD; 7 Medicine, St. George’s University School of Medicine, St. George's, GRD; 8 Medicine and Surgery, St. George's University School of Medicine, St. George's, GRD; 9 Internal Medicine, Clínica Dávila, Santiago, CHL

**Keywords:** post-partum hemorrhage, surgical and nonsurgical treatments, best practices, mortality, prevention, uterine atony, non-pharmacological treatments

## Abstract

Postpartum hemorrhage (PPH) remains the leading cause of maternal mortality worldwide, with uterine atony being the most significant contributing factor. Other risk factors for PPH include increased maternal age, coagulation abnormalities, retained placenta, and prolonged third-stage labor. Despite the potential for prevention through early detection and management, PPH can still occur even in the absence of known risk factors. For this reason, adequate preparation and comprehensive management strategies must be implemented. This study, which comprises research from 2006 to 2023, reviews and analyzes various prevention and management techniques for PPH, including surgical and nonsurgical approaches. Key findings indicate that the presence of well-trained critical control teams is essential for the effective management of PPH. In addition, early detection techniques have significantly reduced mortality outcomes associated with PPH, highlighting their importance in patient care.

## Introduction and background

Postpartum hemorrhage (PPH) is defined as excessive loss of blood that occurs during birth or after birth and is one of the leading causes of mortality and morbidity in the world. In 2017, the American College of Obstetrics and Gynecology redefined PPH as losing more than 1000 ml of blood with cesarean section or 500 ml of blood with vaginal delivery and having signs and symptoms of hypovolemia within 24 hours of delivery regardless of the route of delivery [1]. PPH accounts for 60% of maternal deaths in developing countries, contributing to over 100,000 fatalities globally each year [2,3]. Although the rate of severe maternal mortality differs in both high-income countries and low-income countries, PPH still remains the major cause of maternal mortality worldwide [4]. 

Identifying the risk factors and how to reduce them is essential to prevent PPH. The most common cause of PPH is uterine atony, which occurs in about 80% of cases. Other notable causes include retention of the placenta, prolonged third-phase labor, maternal age above 35 years, previous history of PPH, and coagulation abnormalities such as disseminated intravascular coagulation (DIC) or immune thrombocytopenia (ITP) [2,5,6].

Various interventions such as medications, compression techniques, procedures, and surgeries have been utilized to control the blood loss in PPH [7,8]. Oxytocins increase the uterus' contraction rate and prevent uterine atony. An additional use of oxytocin would be in the acute management of the third stage of labor (AMTSL). This is when oxytocin is used as a prophylaxis to enhance placenta delivery, decreasing the risk of retained placenta and blood loss [9]. Some recommendations and improvements to the gold standard of oxytocin have been introduced, such as administering oxytocin sublingually instead of using the intravenous and intramuscular route to prevent bleeding [4]. Compression techniques such as aortic compression can be used for the expulsion of retained placenta and formed clots. Finally, the retained placenta can be removed manually and followed up with dilation and curettage [7]. The main purpose of this research paper is to analyze and review various medical therapies used in preventing and treating PPH.

## Review

Methods

A rigorous and comprehensive search was done using PubMed, Google Scholar, *American Journal of Obstetrics & Gynecology* (AJOG), *Science Direct*, *American Academy of Family Physicians*, *Reproductive Health*, and SpringerLink. The search was limited to English-language articles that were published from 2006 to 2023. Keywords such as "treatment of postpartum hemorrhage," "prevention and management of postpartum hemorrhage," "maternal mortality," and "uterine atony" were used.

Studies were included if they met the following criteria: 1) studies that were performed or carried out on humans, 2) studies that were published in English, 3) studies published between 2006 and 2023, 4) studies that focused on the prevention and management of PPH, and 5) studies that were published in a peer-reviewed journal. In addition, our systematic review excluded 1) studies focused on animals, 2) studies not focused on preventing and managing PPH, 3) non-peer-reviewed articles, and 4) studies not published in English.

**Figure 1:**
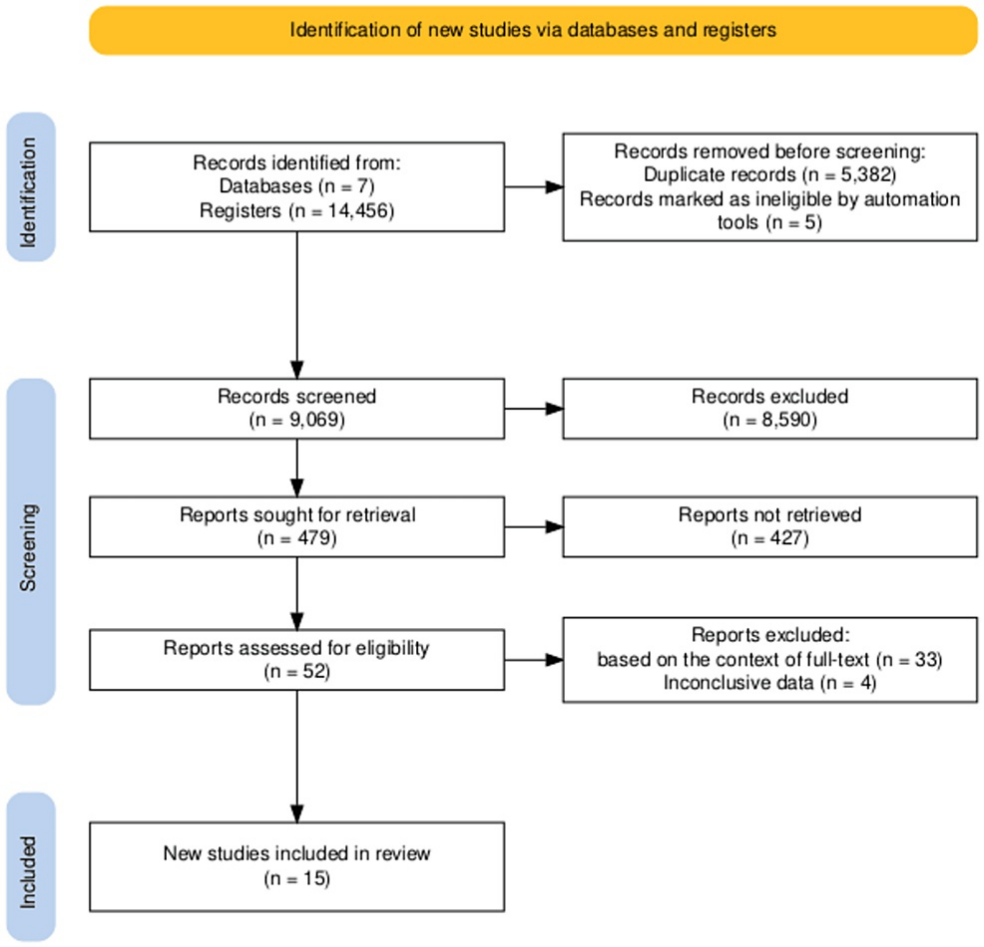
Flow diagram of the selection process based on the study’s inclusion and exclusion criteria The Preferred Reporting Items for Systematic Reviews and Meta-Analyses (PRISMA) diagram was made by the authors.

Three independent reviewers screened the titles and abstracts of all identified records. Thirty-seven articles were removed based on the context of the full text and having inconclusive data. The studies and papers were systematically analyzed, each undergoing a thorough qualitative assessment, as depicted in Figure 1.

Results 

A total of 14,456 publications were found; 3,245 were from PubMed, 4,357 were from Google Scholar, 2,780 were from AJOG, 325 were from Science Direct, and 3,749 were from other databases. The exclusions included 5,382 duplicated publications, and five were removed automatically based on the publication date. A total of 8,590 publications were excluded during the screening process, leaving 479 for retrieval and manual screening. These publications were manually screened based on availability, article type, title, and abstract, leaving 52 articles for eligibility check. Finally, in this paper, 15 articles were used, as shown in Table 1.

**Table 1:** Summary of findings UBT: uterine balloon tamponade, PPH: postpartum-hemorrhage, EBL: estimated blood loss, ICD: International Code for Disease, ICU: intensive care unit, IIAL: internal iliac artery ligation The table was made by the authors.

Authors	Key Findings/Summary	Limitations/Conclusions
Overton et al., 2023 [3]	4,791 patients from 91 studies demonstrated 85.9% efficacy in the resolution of hemorrhage with the use of UBT, with a major success in the setting of atony (87.1%). Vaginal birth registered more success (87.0%) as opposed to cesarean birth (81.7%).	This analysis was shown to be more successful in women that went through vaginal birth than cesarean. The use of UBT for managing hemorrhage should only be used if there’s adequate monitoring of the patient's clinical status after placement. Followed up with immediate access to surgical intervention.
Franke D et al., 2021 [6]	Uterine atony was significantly associated with postpartum hemorrhage (p = 0.048), a decrease in hemoglobin (p < 0.001), an increase in measured blood loss, and the need for blood transfusion.	Early detection of uterine atony is a major factor in the management of postpartum hemorrhage.
Likis FE et al., 2015 [7]	A retrospective cohort study evaluated 91 women with mean age = 33.3 ± 4.6, median parity = 0, range 0-3) undergoing treatment for severe PPH with estimated blood loss of ≥1500 ml within 24 hours after birth. PPH was due to atony in 41.8% of cases. They were treated with intravenous oxytocin (n=33 receiving oxytocin only) and other uterotonic agents (n = 16 receiving oxytocin plus other agents). PPH resolved without the need for additional procedures among the 49 women treated with oxytocin and other uterotonic agents. Uterine atony accounted for 26.5% of cases, while uterine rupture, coagulopathy, and retained placenta accounted for 42.9%.	Bleeding control was achieved without a need for further surgeries in 45 of 91 women receiving oxytocin alone or with other uterotonics. The study does not report analytic comparisons.
Liu C-N et al., 2021 [10]	PPH was observed in 532 mothers among the total population of 34,178 mothers. Abnormal placentation (53.8%) was primarily identified as a cause of PPH, while uterine atony without placental tissues accounted for 38.91% of the cases. The study demonstrated that uterine atony without other causes was lower than the observed prevalence of 70–80%. By contrast, abnormal placentation, which was a significant cause of PPH, was higher than the previously stated prevalence of 10%.	Lack of data on vaginal deliveries and emergency cesarean sessions based on ICD codes.
Callaghan W et al., 2010 [11]	Uterine atony accounted for PPH increased to 26% between 1994 and 2006 from 2.3% (n = 85,954) to 2.9% (n = 124,708; p < 0.001).	Population-based surveillance data demonstrated an apparent increase in PPH caused by uterine atony. Clinical data are needed to understand the factors associated with this trend.
Dueckelmann A et al., 2023 [12]	A study assessed 78 patients with refractory PPH due to atonic bleeding. 60.3% received a chitosan-covered gauze tamponade, whereas 39.7% received a UBT. No significant difference was observed between either group. There was also no difference in the mean estimated blood loss (EBL) (2017 mL gauze; 1756 mL UBT; p = 0.225), admission to ICU, and hemoglobin levels. A controlled study with a larger sample size would be effective to demonstrate the efficacy of this method. This could be explored in low resource settings, as long as adequate monitoring is provided. A full analysis of cost effectiveness should also be assessed with the use of UBT.	Chitosan covered gauze is an excellent option for treating PPH as a result of atony or placental bed bleeding. It is equivalent to balloon tamponade. The study was limited by sample size and its design.
Maria I, 2020 [13]	A feasibility study evaluated the use of mini-sponge tamponade devices in nine patients. This device was made from trauma gauze and was described as successful in the placement and control of PPH. Safety and efficacy of this method in a larger prospective study should be explored. This should be in comparison with alternate methods.	More data will be required to evaluate the safety and efficacy of this technique.
Doumouchtsis et al., 2014 [14]	A systematic review evaluated 125 women who had been treated for postpartum hemorrhage by uterine compression sutures. They estimated that 91% of the women have normal menstrual cycles by six months after their compression procedure and that 75% of those who desired another pregnancy achieved conception. The results show that this technique does not alter subsequent fertility. Although the data on this are limited, it deserves to be explored more by further studies, with higher statistical power. A better research and information regarding fertility after this procedure, and its associated risks could relieve potential mothers of their worries regarding a new pregnancy.	Uterine surgical techniques for the management of severe PPH have no adverse effect on menstrual and fertility outcomes in most women. However, the number of studies and the quality of evidence available are limited.
Shahinoor et al., 2022 [15]	Forty-five patients from three different African countries participated in research implementing preventative strategies such as E-MOTIVE to reduce PH. This includes multidisciplinary simulation training, which improves team communication and collaboration to improve early detection of PH.	Inadequate training of healthcare providers, especially in developing countries, is a major contributor to the early detection and emergency treatment of PH.
Kayem G et al., 2011 [16]	A total of 211 women were treated with a uterine compression suture to control postpartum hemorrhage. The effectiveness rate was 75% (95% confidence interval (CI), 69–81), and the method of compression technique used (compress or multiple sutures, B-Lynch) showed no difference in the outcome of hysterectomy. This is a simple procedure that could be tried before any complex surgery with a shorter operating time. There’s the risk of intrauterine adhesions and scarring after this procedure. This possibilty has not yet been explored. It could be beneficial if assessed in future studies especially for women who desire another pregnancy.	This technique is majorly used in PPH cases caused by uterine atony and refractory to medical treatment.
Kaya B, 2017 [17]	Among 26 women with uterine atony, 12 had primary and 14 secondary procedures of IIAL. Following bleeding after a B-Lynch uterine compression procedure. There was a success rate of 87% with this procedure. This is a difficult procedure that requires thorough knowledge of pelvic vascular anatomy and exposure. It can cause severe morbidity like iliac vein injury, gluteal claudication, and ligation of ureter. This should only be assessed in major surgeries.It should be advised that this procedure is not often performed as it requires obstetricians that have less experience in surgery.	It is important to propose second-line surgical treatment after failure of ligation or uterine compression sutures to stop PPH hemorrhage. This should be offered before performing a peripartum hysterectomy.
Sentilhes L, 2008 [18]	B-Lynch sutures controlled hemorrhage and avoidance of hysterectomy in 12 of 15 cases (80%) of women with PPH after cesarean deliveries despite vessel ligation. There is the risk of uterine necrosis following B-Lynch suture, resulting in subsequent hysterectomy in a patient. It is important for future studies that sufficient data are collected on the patient population before employing this procedure as the first-line surgical treatment of PPH.	The B-Lynch technique has shown to be an effective procedure with a low morbidity in the control of severe PPH following a failure of vessel ligation.
Purwosunu et al. 2016 [19]	A preliminary study on vacuum-induced uterine tamponade demonstrated bleeding control in 10 patients who failed first-line PPH treatment following vaginal birth. They had an EBL of 600 to 1000 mL before placement of the device. Evacuation of 50 to 250 mL of blood and hemorrhage control was achieved in <2 minutes. A large-scale, prospective data will be needed to establish the safety and efficacy of this device in future. The optimization of this device should also be explored further, as it can be assessed in terms of device efficacy, when used with compression sutures technique and uretonics. The cost of this device in comparison to other methods should also be put into consideration.	This investigation proposes that a device made to create vacuum-induced uterine tamponade may be a better alternative in the treatment of atonic postpartum hemorrhage. This device is contraindicated in women with abnormal uterine anatomy and intrauterine infection.
D’Alton et al. 2020 [20]	A successful treatment was observed in 94% (100/106, 95% CI 88-98%) of 106 participants that participated in a treatment with vacuum-induced tamponade. One hundred had successful bleeding control at a median of three minutes and a median EBL during treatment of 110 mL after vacuum connection. This device demonstrated a >90% success rate in the control of abnormal bleeding. This can be beneficial in future studies, as it can be incorporated in a common treatment goal, thereby improving maternal recovery.	This method of hemorrhage control may provide a rapid and effective treatment option for postpartum hemorrhage, with the potential to prevent maternal morbidity and mortality. This device will be ineffective if cervical dilation is less than 3 cm.
Goffman et al. 2023 [21]	An observational study of 800 individuals (n = 530 vaginal births, n = 270 cesarean births) who were treated with an Intrauterine vacuum-induced hemorrhage control device, of whom 94.3% had uterine atony. A treatment success rate was recorded as 92.5% for vaginal births and 83.7% for cesarean births. The median indwelling time was 3.1 hours and 4.6 hours, respectively. Bleeding control was achieved in ≤5 minutes for 73.8% in the vaginal and 62.2% post cesarean. There was no record of uterine perforations or deaths. In a highly structured controlled clinical trial that assesses data collection and patient management, arriving at similar conclusions will be unattainable. There would be difficulty in ascertaining data such as blood loss volume and time of hemostasis in real-life treatment of PPH. This is because blood loss in this study was estimated and most likely underestimates the total amount of blood loss. The study was intended to assess use of the device; therefore, comparisons to future treatments would be futile.	This device is important in managing a life-threatening condition, and timely utilization may help improve obstetric hemorrhage outcomes.

Research has shown that there has been a significant reduction in the rate of PPH, especially due to uterine atony. The use of less invasive interventions, such as IV oxytocin, resulted in adequate bleeding control without the need for any surgical procedures. Refractory PPH can be managed with uterus-preserving techniques, such as internal iliac artery ligation. This has shown to be successful without any prospective surgical intervention. Furthermore, due to limited resources in developing countries, mini-sponge tamponade devices were not shown to be inferior to UBT. It can be used in place of this device as it is readily available in emergencies.

Although PPH is mainly caused by uterine atony, the majority of the studies explored have identified ways of preventing it. There have not been a lot of studies directed toward preventing PPH caused by abnormal placentation. This lack of data may contribute to inadequate management of PPH caused by other risk factors. We can infer from the table that past and recent studies have shown interventions to treat PPH generally proceed from less to more invasive procedures, including medications, intrauterine devices, surgeries, and compression techniques.

Discussion

Maternal mortality is a major issue the healthcare system has been dealing with for decades. The advancement of medicine must keep improving on how to deal with the fatalities associated with childbirth effectively. Different methods have been explored, and the development of heat-labile oxytocin tablets conveys the current standards of medicine, which is a patient-centered care system [3]. Postpartum women in certain geographical areas have limited access to trained healthcare workers for the administration of oxytocin via intravenous or intramuscular routes. Thus, to reduce the mortality rates of these postpartum women, a readily available pill that can be administered sublingually has been proposed [3]. 

Prevention medicine is still the best approach for dealing with maternal mortality post-childbirth, and this is why thorough history-taking becomes essential. Effective history gathering can detect pregnant women at risk of experiencing immune thrombocytopenia (ITP) before delivery. One recommendation for ensuring a better outcome for pregnant women at risk of experiencing ITP is the administration of prednisone (1 mg/1 kg) [5]. This is considered the first-line treatment and is given when platelet levels reach unsatisfactory levels of approximately 30,000/μL or when there are signs and symptoms of bleeding in the pregnant woman, such as petechiae, epistaxis, and purpura. If the treatment with prednisone is unsuccessful, intravenous gamma globulin (IVIg) can be administered (usually 2 gm/kg administered in divided doses over two to five days) [5]. The advantage of administering IVIg is to prevent splenectomy in the pregnant woman. In rare cases where there is refractory ITP, post IVIg administration, concurrent treatment with a high-dose IV corticosteroids (such as methylprednisolone 1g IV) is recommended. Oxytocin and ergometrine are the traditional first-line approaches to achieving contraction in the case of uterine atony. However, ergometrine should be avoided in preeclamptic patients due to its hypertensive effects [5]. If the uterus remains atonic after delivery of the placenta, methylergometrine can be given intramuscularly [22].

It is imperative to be aware that every birth delivery process has a chance of leading to hemorrhage. It has been estimated that 20% of PPH occurs in women with no risk factors. Therefore, physicians should be prepared to manage this condition in every labor process [23]. Other risk factors for PPH include preeclampsia, multiple gestation, maternal obesity, fetal macrosomia, prolonged labor, augmented labor, and primiparity [8,23]. Response strategies should be implemented to deal with postpartum emergencies. A response team that gets alerted during a PPH should be established, and this team needs education on how to activate the emergency release of transfusion protocols and run drills from time to time. The availability of a hemorrhage cart equipped with supplies, a checklist, and instruction cards is vital [23]. 

A major strategy for preventing PPH is being sensitive and culturally inclined to people’s beliefs. For instance, Jehovah's Witnesses are known to refuse blood transfusion if needed, and it is essential to have an agreement on the treatment plan and the possibility of eliminating routine episiotomy. The best preventive strategy is active management of the third stage of labor, which includes administering a uterotonic drug with, or soon after, the delivery of the anterior shoulder and controlled cord traction [24]. The International Federation of Gynecology and Obstetrics (FIGO) defines active management as the third stage of labor, which involves delaying cord clamping and uterine massage after delivery of the placenta. Delaying cord clamping for about 60 seconds increases iron stores and decreases anemia, which is especially important in preterm infants and low-resource settings [24].

Another major cause of postpartum mortality is uterine atony. To impede this, the techniques performed are external uterine massages and bimanual compressions. The compression technique aids in the removal of retained placenta or clots. Other helpful management consists of manual removal of the placenta, uterine balloon tamponade, uterine artery embolization, and manual removal of blood clots. When PPH is due to trauma to the genital tract, a laceration repair is warranted. If all these measures are ineffective at controlling the bleeding, there are surgical options like curettage, uterine compression sutures, hysterectomy, and uterine and pelvic artery ligation [7,8]. Intrauterine balloon tamponade is highly effective. The catheter is readily available and inexpensive and does not require extensive training for insertion, which leads to the avoidance of major surgery [25].

## Conclusions

The complications of PPH are very common, even in developed countries with highly trained employees or fully staffed delivery facilities. Thus, for a major decline in mortality rates associated with PPH to happen, hospitals or birthing centers must implement protocols that not only detect the early signs of PPH but also be fully equipped to take action to prevent it. One of the discussed protocols is the availability of a hemorrhage cart. This hemorrhage cart must be checked daily or at the beginning of each shift.

Other viable options for the prevention and treatment of PPH are efficient history taking from pregnant women, having a solid treatment plan, and better management of the third stage of labor, which consists of the use of uterotonic medications, delaying cord clamping, and uterine massage after delivery of the placenta. External uterine massages, aortic compressions, use of uterine balloon tamponade, manual removal of blood clots and placenta, and uterine artery embolization were also reviewed. Further exploration of the proposed sublingual oxytocin tablets would also benefit and save many lives, most especially in countries that lack access to advanced healthcare resources.
